# A Role of CREB in BRCA1 Constitutive Promoter Activity and Aromatase Basal Expression

**Published:** 2008-12

**Authors:** Sagar Ghosh, Yunzhe Lu, Yanfen Hu

**Affiliations:** *Department of Molecular Medicine/Institute of Biotechnology, University of Texas, Health Science Center at San Antonio, USA*

**Keywords:** aromatase, BRCA1, CREB

## Abstract

Aromatase is the rate-limiting enzyme in estrogen biosynthesis and a key target in breast cancer treatment. Its ovary-specific promoter, pII, is induced in response to protein kinase A (PKA) activation. It has been proposed that breast cancer susceptibility gene 1, BRCA1, is involved in negative regulation of aromatase pII activity. Surprisingly, inhibition of PKA pathway by inhibitor H89 elevates basal aromatase expression while abolishes cAMP-mediated aromatase induction in an ovarian granulosa cell line, KGN. In this report, we decipher the mechanism by which the PKA pathway negatively regulates aromatase basal expression. We show that PKA pathway plays a positive role in the expression of BRCA1. H89 effectively reduces endogenous BRCA1 mRNA levels as well as reporter gene expression from a BRCA1 promoter. Mutation of a cAMP-responsive element (CRE) in the BRCA1 promoter reduces BRCA1 expression. Chromatin immunoprecipitation (ChIP) shows that CRE-binding protein, CREB, binds to the BRCA1 promoter. Furthermore, knockdown of CREB in KGN cells leads to decreased BRCA1 level as well as elevated basal aromatase mRNA expression. These data demonstrate that both the CRE site in the BRCA1 promoter and CREB are required for BRCA1 constitutive expression. Our study suggests that PKA pathway exerts its negative impact on basal aromatase expression indirectly by contributing to the constitutive expression of BRCA1.

## INTRODUCTION

Aromatase P450 (CYP19) is the key enzyme in estrogen biosynthesis and there is a growing awareness that aromatase plays a significant role in breast cancer development (1, 2). In premenopausal women, aromatase is predominantly expressed from an ovary-specific promoter (pII) in response to gonadotropins such as follicle stimulating hormone (FSH) and leutinizing hormone (LH). It is believed that FSH and LH induce pII promoter activity by activating protein kinase A (PKA) pathway (3). The induction of aromatase in response to FSH/LH during ovarian cycles is relatively well characterized. However, little is known about the control of aromatase basal expression. The elevated aromatase basal expression may play a more prominent role in postmenopausal breast cancer development when gonadotropins no longer orchestrate estrogen cycles. Furthermore, intratumoral aromatase activity is increased in postmenopausal breast cancer, likely resulting from elevated basal pII promoter activity (2). It is therefore highly important to understand the control of basal aromatase expression in addition to the mechanism of FSH-mediated aromatase induction.

## MATERIALS

### Cell Lines and Drugs

Human ovarian granulosa cell line KGN was a gift from Dr. Hajime Nawata (Kyushu University, Japan) and has been previously described (4). Forskolin (Cat # F6886) was purchased from Sigma. H89 (Cat # 371963), PD98059 (Cat # 513000) and Wortmannin (Cat # 681675) were purchased from Calbiochem while Rapamycin (Cat # R-1018) was purchased from A.G. Scientific. Inc.

### Antibodies and Immunoblotting kit

Antibodies against Aromatase (Serotec, MCA-2077), BRCA1 (Oncogene Ab-1/OP-92), α-tubulin (Calbiochem CP06), ATF1 (Santa Cruz Biotechnology sc-186) and CREB (Santa Cruz Biotechnology sc-186X) were purchased from corresponding commercial sources. All immunoblots were developed using an ECL kit from Pierce (#34080).

### Real-time PCR Primers and Small Interfering RNA (siRNA) Duplexes

The sequences of the specific PCR primers are:

hArom-2F, 5’TGGAATTATGAGGGCACATCC3’;

hArom-3R, 5’GTCCAATTCCCATGCAGTAGC3’;

hBRCA1Ex20-F, 5’CCAAAGCGAGCAAGAGAATCC3’;

hBRCA1Ex21-R, 5’TGAAGGGCCCATAGCAACAG3’;

huGAPD69f, 5’CCATCAATGACCCCTTCATTG3’;

huGAPD154r, 5’GACGGTGCCATGGAATTTG3’;

NR4A2 prom F, 5’CCACCCAAGCTGGCTACCAA3’;

NR4A2 prom R, 5’GTTTATGTGGCTTGCGCTGC3’;

BRCA1 prom F, 5’TTTCCTTTTACGTCATCCGGG3’;

BRCA1 prom R, 5’GCTAAGCAGCAGCCTCTCAGA3’;

BRCA1 Downstream F, 5’ACTGGCCAACAATTGCTTGACT3’;

BRCA1 Downstream R, 5’AGACCCTTACCCAATTCAATGTAGA3’;

GAPDH prom F, 5’GAGAAAGTAGGGCCCGGCTA3’;

GAPDH prom R, 5’GGTCTTGAGGCCTGAGCTACG3’.

The siRNA duplexes were purchased from Dharmacon (CREB D-003619-03, CREB D-003619-07, ATF1 D-010045-05)

### Plasmids

The wildtype proximal BRCA1 promoter (L6), and the USCAAT deletion were generated by PCR and cloned into pGL3-basic luciferase reporter vector (Promega).

BRCA1-prom-L6-F, 5’TCTacgcgtgAATTCTTCCTCTTCCgTCTCTTTC3’;

BRCA1-prom-L6-R, 5’TATAgATCTgAgCTCACgCCgCgCAgTCgC3’;

BRCA1-prom-USCAAT-F, 5’TCTACgCgTgACTgggTggCCAATCCAgAg3’.

## METHODS

### RT-PCR

mRNA levels were measured by real-time RT-PCR. Total RNA was isolated using TRIzol Reagent (Invitrogen) according to manufacture’s instructions. RNA was reverse-transcribed using the ImPrompII kit from Promega. SYBR Green-based real time PCR assay was conducted following the manufacturer’s procedures (Applied Biosystems for ABI7300). GAPDH was used for normalizing the real time PCR results. The data is expressed as a fold (Aromatase mRNA), or a percentage (BRCA1 mRNA) relative to the DMSO-treated control.

### Transfection and Luciferase Assay

For the dual-luciferase assay, 0.8 × 10^5^ KGN cells were transiently co-transfected using Fugene 6 (Roche) with 0.5μg of the indicated reporter construct, and 1 ng of a control phRL-SV40-renilla reporter construct for normalization of transfection efficiency. 6 hours post-transfection, the cells were treated with DMSO or 20 μM H89, 30 hours post-transfection the cells were lysed and assayed using the dual-luciferase reporter assay system (Promega). The relative luciferase activity of each deletion construct is expressed as a percentage of proximal L6 promoter construct.

### Chromatin Immuno-precipitation (ChIP)

The ChIP experiment was performed following a previously described protocol (5). Briefly, 1 × 10^7^ KGN cells were crosslinked with 1% formaldehyde (Sigma Cat # 252549) for 8 min, followed by termination of the crosslinking with 0.125 M glycine. Cell lysates were prepared and chromosomal DNA was sonicated to reach average sizes between 500-1000 bp. The lysate was immunoprecipitated with either rabbit IgG or an anti-CREB antibody. Upon reversal of the crosslinking, the purified DNA fragments were PCR-amplified for proximal and distal regions of aromatase pII (6), BRCA1 and NR4A2 promoters.

### siRNA knockdown

CREB proteins were knocked down following a protocol described in our earlier paper (7).

## RESULTS

**Figure 1:**
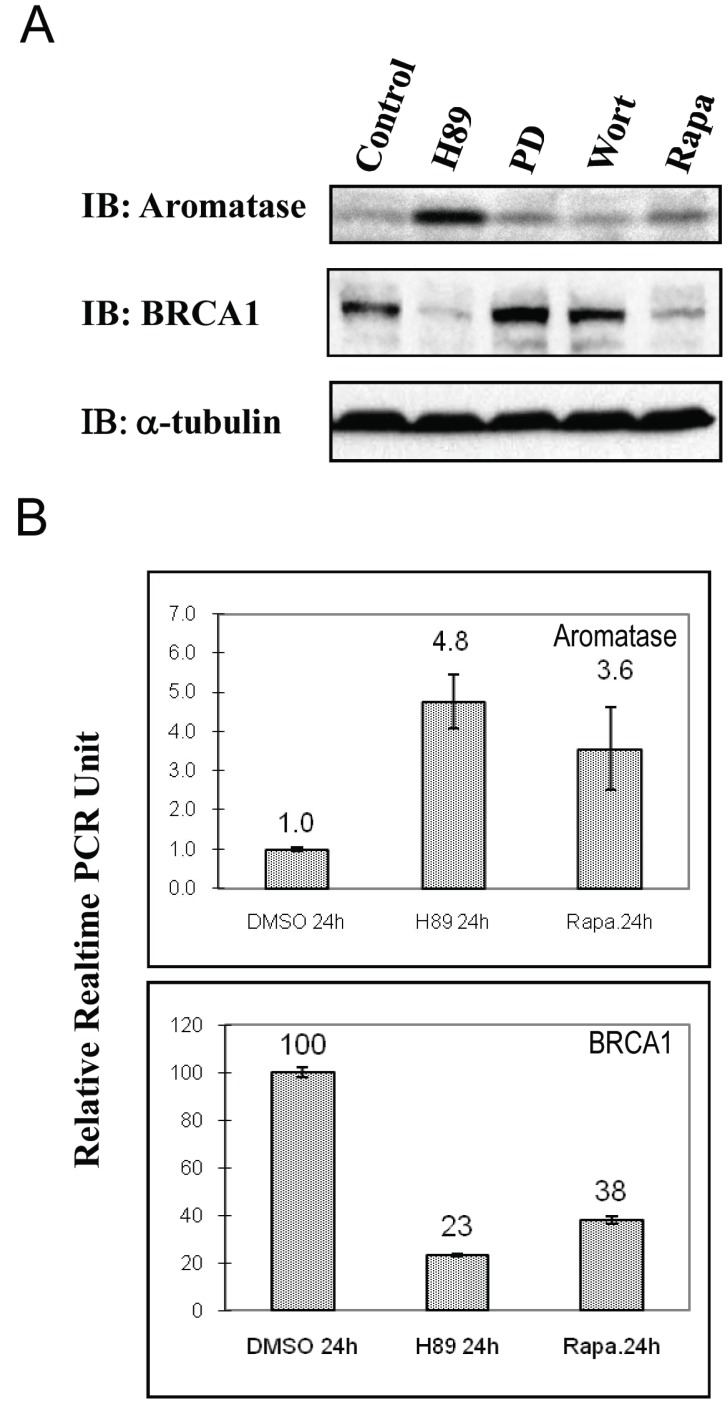
Effect of H89 on aromatase and BRCA1 expression in KGN cells. A, The human ovarian granulosa cell line KGN were treated with control (DMSO), 20 mM H89, 50 mM PD98059, 200 nM Wortmannin, 100 nM Rapamycin 100 nM, and cells were harvested 24 hours after treatment. Immunoblotting was performed using specific antibodies against aromatase, BRCA1 and α-tubulin; B, Aromatase and BRCA1 mRNA levels were measured by real-time RT-PCR. Gapdh was used for normalizing the real time PCR results. The data are expressed as a fold (Aromatase mRNA), or a percentage (BRCA1 mRNA) relative to the DMSO-treated control.

Our previous study using an ovarian granulosa cell line KGN showed that the breast tumor suppressor BRCA1 is involved in down-regulating aromatase expression (8). Forskolin, which elevates cAMP level in cells and activates the PKA pathway, markedly reduced BRCA1 level in accordance with aromatase induction (8). To test whether PKA activation is required for forskolin-mediated BRCA1 reduction and aromatase induction, a PKA inhibitor, H89, was included in the forskolin treatment. As expected, H89 abolished forskolin-mediated aromatase induction (Lu, unpublished data). Surprisingly, instead of preventing PKA-induced BRCA1 level reduction, H89 significantly reduced basal BRCA1 levels in the absence of forskolin treatment (Fig. 1A) (9). A time course conducted over 24 hours showed that BRCA1 mRNA levels gradually decreased in the presence of H89 (Lu, data not shown). We have previously shown that knockdown of BRCA1 mRNA by small interferring RNA (siRNA) led to elevated basal aromatase expression in KGN cells (8). The marked reduction of BRCA1 by H89 prompted us to examine the effect of H89 on aromatase level. Interestingly, H89-mediated BRCA1 reduction also leads to increased basal aromatase expression (Fig. 1A, lane 2). An mTOR inhibitor, rapamycin, displayed similar effects as H89 (Fig. 1A, lane 5). The other kinase inhibitors, MEK inhibitor PD98059 and PI3 kinase inhibitor wortmannin, had no effect on BRCA1 mRNA levels (9) and consistently, aromatase expression was unchanged (Fig. 1A, lanes 3 & 4). The decrease in BRCA1 and elevation of aromatase protein levels predominantly resulted from changes in BRCA1 and aromatase mRNA levels, as real-time PCR indicated that BRCA1 mRNA levels were decreased by 5- and 3-fold while aromatase mRNA levels were increased by 4.8- and 3.6-fold, upon H89 or rapamycin treatment, respectively (Fig. 1B).

**Figure 2:**
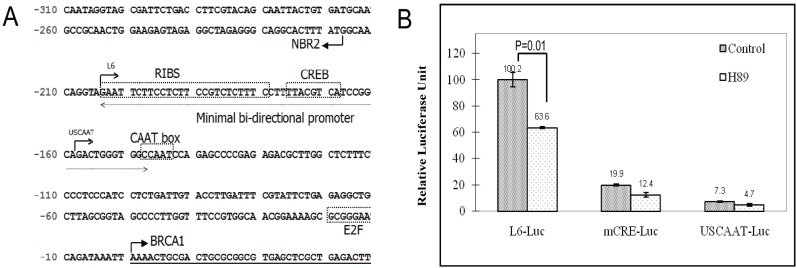
H89 targets the CRE in BRCA1 promoter. A, The nucleic acid sequence of the BRCA1 proximal promoter; B, The activities of wildtype proximal BRCA1 promoter reporters (L6-Luc) is reduced by H89 treatment, and the mutant promter reporters without functional CRE (mCRE-Luc and USCAAT-Luc) abolish BRCA1 promoter activity.

It has been reported that *BRCA1* proximal promoter contains a cyclic-AMP (cAMP)-responsive element (CRE) between -176 & -169 base pairs (bp) that is important for the constitutive expression of the promoter in MCF-7, T47D and HepG2 cells (Fig. 2A) (10). To test whether reduced BRCA1 mRNA level upon H89 treatment reflects decreased *BRCA1* promoter activity, a DNA fragment corresponding to the *BRCA1* promoter -204 to +27 was fused to a luciferase gene and the resultant luciferase reporter construct (L6-Luc) was transfected into KGN cells. As shown in Fig. 2B, the luciferase activity of L6-Luc reporter, like the endogenous BRCA1 mRNA level, decreased upon H89 treatment, suggesting that H89 reduces BRCA1 mRNA by reducing BRCA1 promoter activity. Furthermore, consistent with previous observation in several breast cancer cell lines (10), the CRE site is important for BRCA1 expression in ovarian granulosa cells, as both point mutation (mCRE-Luc) and deletion of the CRE site (USCAAT-Luc) abolished luciferase activity.

**Figure 3:**
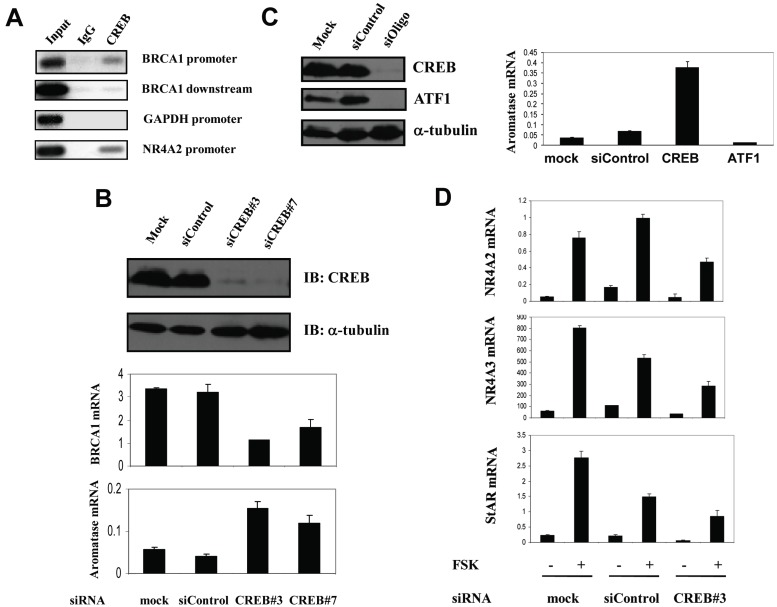
CREB regulates BRCA1 promoter. A, CREB is present in BRCA1 promoter in chromatin immunoprecipitation assay; B, Knockdown of CREB by siRNA leads to reduced BRCA1 and elevated aromatase basal expression; C, Aromatase basal expression is not affected by ATF1 knockdown; D, In contrast to aromatase gene, CREB knockdown reduces expression of NR4A2, NR4A3 and StAR, which are known CREB target genes.

The pivotal role of CRE in *BRCA1* promoter activity prompted us to examine whether the CREB protein, a well characterized transcription factor that binds to CRE site, plays a role in *BRCA1* promoter activity. Chromatin immunoprecipitation (ChIP) assay using CREB-specific antibody revealed that CREB is indeed present in the *BRCA1* promoter, but absent downstream of BRCA1 gene, as CREB ChIP signal using primers from downstream of *BRCA1* gene was similar to that of a control IgG ChIP (Fig. 3A). CREB ChIP signal on the promoter of another steroidogenesis gene, NR4A2, served as a positive control (Fig. 3A) as it is well established that CREB binds to, and regulates, the *NR4A2* promoter (11-13). To further test whether CREB is functionally important for BRCA1 expression, siRNA against CREB was employed to knockdown CREB mRNA in KGN cells. As shown in Fig. 3B, two independent siRNA duplexes effectively reduced CREB mRNA levels by 90%. Interestingly, knockdown of CREB indeed decreased the endogenous BRCA1 mRNA level while increased the aromatase basal level (Fig. 3B). Furthermore, knockdown of another CREB family member ATF1 does not appear to have had the same effect on aromatase expression (Fig. 3C), suggesting that CREB might be a major player in BRCA1 promoter activity. It has been well established that CREB is a pivotal activator in several steroidogenic genes such as NR4A1, NR4A2 and StAR (14-16). Consistent with that notion, knockdown of CREB also abolished forskolin-induced expression of NR4A1, NR4A2 and StAR (Fig. 3D), suggesting the effect on aromatase expression by CREB knockdown is promoter-specific.

## DISCUSSION

**Figure 4:**
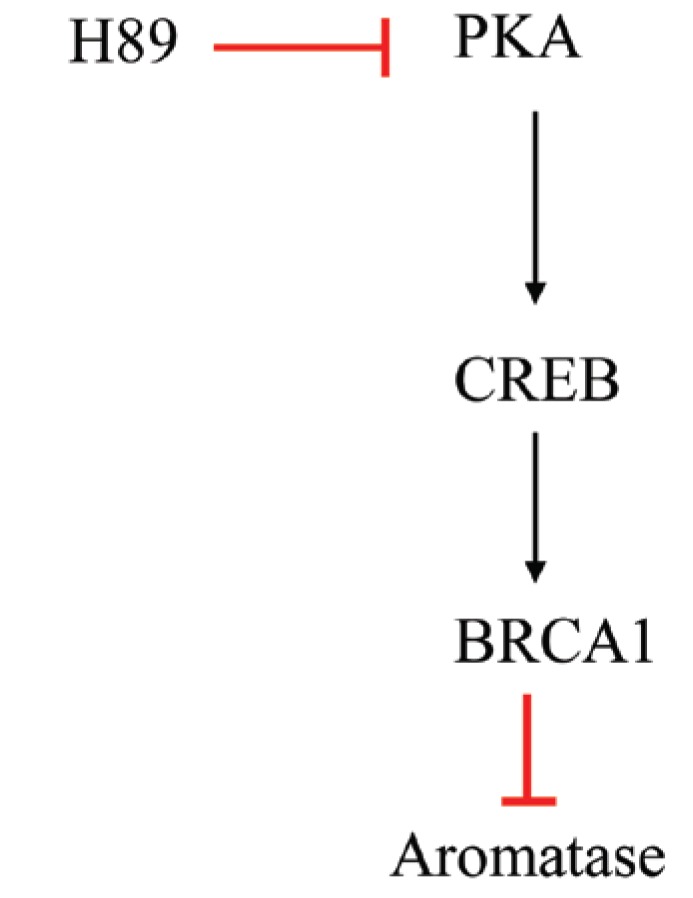
A model for H89 effect on BRCA1 and aromatase expression.

We have previously reported that knockdown of BRCA1 in ovarian granulosa cells results in elevated aromatase basal expression, providing a molecular explanation for why loss of BRCA1 predominantly leads to breast and ovarian cancers in women (8). We report here an intriguing observation that a PKA inhibitor, H89, also significantly reduces BRCA1 mRNA levels while elevating basal levels of aromatase mRNA. Thus, this study provides an independent approach addressing the relationship between BRCA1 and aromatase expression. In addition, we identified CREB as the protein required for constitutive BRCA1 promoter activity. We propose that H89 inhibits CREB activity which in turn results in decreased BRCA1 expression. The reduced BRCA1 expression would then lead to elevated aromatase basal expression (Fig. 4).

Aromatase promoter activity has been extensively studied in the context of FSH/LH induction. It is clear that the cAMP-PKA pathway plays a major role in aromatase induction. It has been proposed that CREB activates aromatase expression by binding to the CLS element in the aromatase pII promoter. So far, the evidence for involvement of CREB at the aromatase promoter is limited to *in vitro* gel retardation assay. Direct *in vivo* evidence is still missing. We attempted CREB ChIP on the aromatase promoter, but were not able to detect any CREB binding on the endogenous aromatase pII promoter (Ghosh, unpublished data). On a related note, a genomewide search of CREB targets by microarray of ChIP DNA (ChIP-chip) using CREB antibody revealed NR4A as one of the potential target, but not the aromatase gene (11-13). It is possible that other members of CREB family are responsible for cAMP-mediated aromatase induction. In this study, we report that CREB plays an indirect role in aromatase expression. We show that CREB negatively regulates aromatase basal expression by maintaining constitutive BRCA1 level in ovarian granulosa cells. Compared with cAMP-induced aromatase induction, regulation of the aromatase promoter in the absence of FSH stimulation has not been fully characterized. However, elevated basal estrogen levels could increase the risk of breast and ovarian cancer, as observed in postmenopausal breast cancers and women undergoing hormone replacement therapy. Therefore it is equally important to characterize FSH-dependent and -independent regulation of aromatase promoter in order to shed lights on different pathways in breast cancer development.

## References

[1] Simpson ER (2002). Aromatase-a brief overview. Annu. Rev. Physiol.

[2] Sasano H, Harada N (1998). Intratumoral aromatase in human breast, endometrial, and ovarian malignacies. Endocrine Rev.

[3] Richards JS (1994). Hormonal Control of Gene Expression in the Ovary. Endo. Reviews.

[4] Nishi Y (2001). Establishment and characterization of a steroidogenic human granulosa-like tumor cell line, KGN, that expresses functional follicle-stimulation hormone receptor. Endocrinol.

[5] Aiyar SE (2004). Attenuation of estrogen receptor alpha-mediated transcription through estrogen-stimulated recruitment of a negative elongation factor. Genes & Dev.

[6] Ghosh S (2005). Jun proteins modulate the ovary-specific promoter of aromatase gene in ovarian granulosa cells via a cAMP-responsive element. Oncogene.

[7] Ghosh S (2007). Tumor suppressor BRCA1 inhibits a breast cancer associated promoter of the aromatase gene (CYP19) in human adipose stromal cells. Am. J. Physiol. Endocrinol. Metab.

[8] Hu Y (2005). Modulation of aromatase expression by BRCA1: a possible link to tissue-specific tumor suppression. Oncogene.

[9] Lu Y (2007). Ubiquitination and Proteasome-Mediated Degradation of BRCA1 and BARD1 during Steroidogenesis in Human Ovarian Granulosa Cells. Mol. Endocrinol.

[10] Atlas E, Stramwasser M, Mueller C (2001). A CREB site in the BRCA1 proximal promoter acts as a constitutive transcriptional element. Oncogene.

[11] Conkright MD (2003). Genome-Wide Analysis of CREB Target Genes Reveals A Core Promoter Requirement for cAMP Responsiveness. Mol. Cell.

[12] Impey S (2004). Defining the CREB regulon: a genome-wide analysis of transcription factor regulatory regions. Cell.

[13] Zhang XK (2005). Genome-Wide analysis of cAMP-responsive element binding protein occupancy, phosphorylation, and target gene activation in human tissues. Proc. Natl. Acad. Sci. USA.

[14] Maruyama K (1998). The NGFI-B subfamily of the nuclear receptor superfamily (Review). Int. J. Oncol.

[15] Herschman HR (1991). Primary response genes induced by growth factors and tumor promoters. Annu. Rev. Biochem.

[16] Wu Y (2005). The orphan nuclear receptors NURR1 and NGFI-B modulate aromatase gene expression in ovarian granulosa cells: a possible mechanism for repression of aromatase expression upon luteinizing hormone surge. Endocrinology.

